# The Legacy of Hermann Rorschach and His Inkblots: Portrait of a Man or Mental Disease?

**DOI:** 10.7759/cureus.67805

**Published:** 2024-08-26

**Authors:** Taylor L Shantz, John C Cravero, Megan Newman

**Affiliations:** 1 Psychiatry, Texas A&M College of Medicine, Bryan, USA; 2 Internal Medicine, Baylor Scott & White Medical Center - Temple, Temple, USA

**Keywords:** medical history, history of psychiatry, inkblots, hermann rorschach, historical vignette

## Abstract

Hermann Rorschach was a Swiss psychiatrist and psychoanalyst who experimented with inkblots for the purpose of diagnosing mental illness and personality temperaments. This paper highlights the legacy of Rorschach through discussion of the events that led to the inkblots' creation during Rorschach’s life and after his death. The foundational elements of the inkblots were conceived in Rorschach’s 1911 dissertation regarding reflex hallucinations, a form of synesthesia, that ultimately served as Rorschach’s experimental focus and development of his iconic inkblots. After Rorschach’s death in 1922, the inkblots were disseminated in America by Samuel Beck and Bruno Klopfer during the 1930s and 1940s while expanding on intelligence and personality metrics. Further research regarding the concept of a “group Rorschach'' was expanded by Molly Harrower and ultimately applied by Douglas Kelly and Gustav Gilbert to the Nazi Defendants at Nuremberg with the aim of conceptualizing the “Nazi personality.” However, due to interpreter bias and conflicting interpretation, the results surrounding the Nazi Rorschachs remain controversial. Further controversy ensued after John Exner’s attempt to standardize the Rorschach methodology while introducing new diagnostic metrics. Today, the Rorschach inkblots are criticized for their lack of validity and clinical relevance.

## Introduction and background

The term ‘Rorschach’ has become synonymous with psychological testing. First developed by the Swiss psychiatrist Hermann Rorschach, the Rorschach inkblot method was a relatively simple test involving 10 cards, each with a single enigmatic and symmetrical inkblot designed to evoke a response for the purpose of psychological analysis and introspection. Rorschach’s creation of the inkblots coincided with the rise of Freudian psychoanalytic theories as well as events that revolutionized the field of psychiatry by his mentors, which included Carl Jung and Eugene Bleuler [1]. Jung sought to reveal a subject’s unconscious through associations between verbal stimuli through his famous Word Association Test [2]. Bleuler invented several neologisms that included the term schizophrenia, which had previously been called dementia praecox [1]. It was through this paradigm that Rorschach investigated the use of 10 inkblots to serve as associative stimuli allowing him to introspectively analyze a subject’s affective and cognitive state, while also maintaining an emphasis on schizophrenics, who not only comprised over half of Rorschach’s experimental test subjects, but also gave the most intriguing responses [1]. 

While these 10 inkblots have remained unchanged since their creation, they have a unique history all to themselves that reflect not only the life and purview of their paternal creator, but also the influence of several independent investigators after Rorschach’s death. In order to appreciate the legacy of Rorschach, the purpose of this paper shall be twofold: first, to explicate the details of Hermann Rorschach’s life and how they contributed to the inkblots' unique design and methodology, and second, to underscore the rise and fall of the Rorschach test throughout notable epochs within the 20th century.

The biographical and historical events described in the following manuscript were obtained from various source material. Several articles were obtained from PubMed after searching terms ‘Rorschach’ and ‘History.’ Meta-analysis studies regarding Rorschach were also obtained from PubMed by search terms ‘Rorschach’ and “meta-analysis.’ Our analysis also includes material obtained from various first and secondhand sources, such as textbooks and biographies.

Hermann Rorschach: a biographical sketch

**Figure 1:**
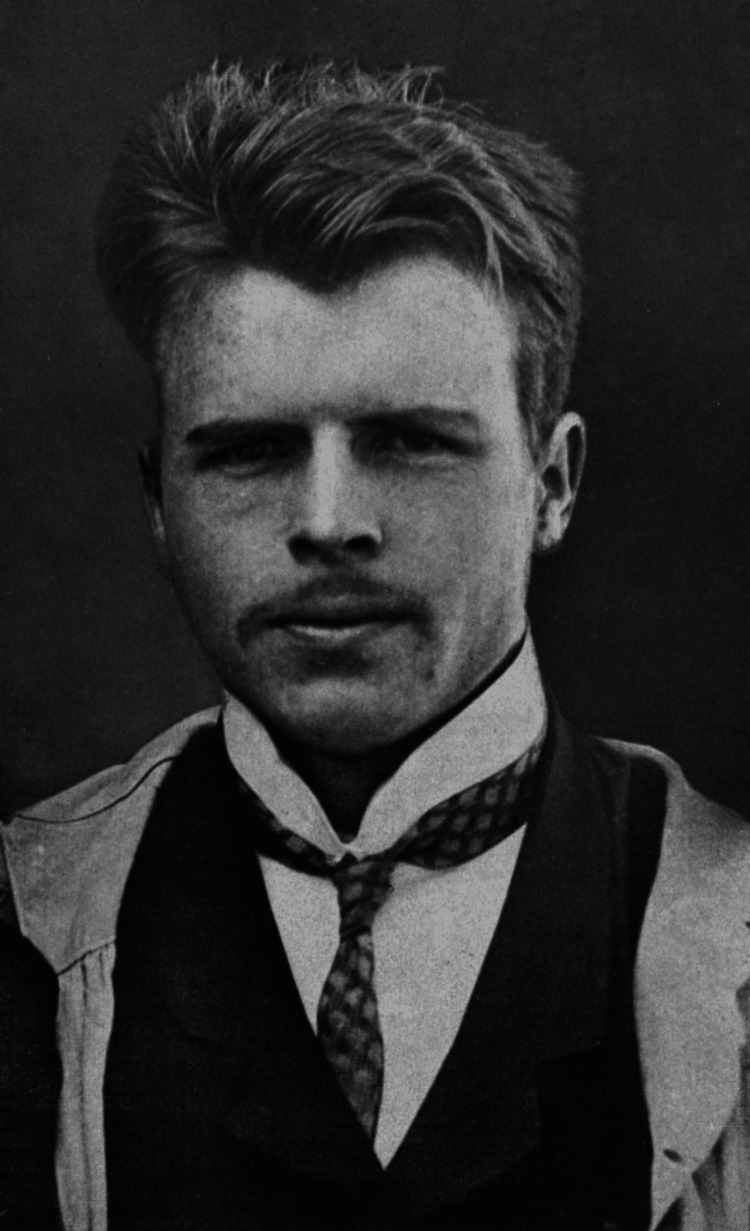
Hermann Rorschach Image available on Wikimedia as part of public domain

**Figure 2:**
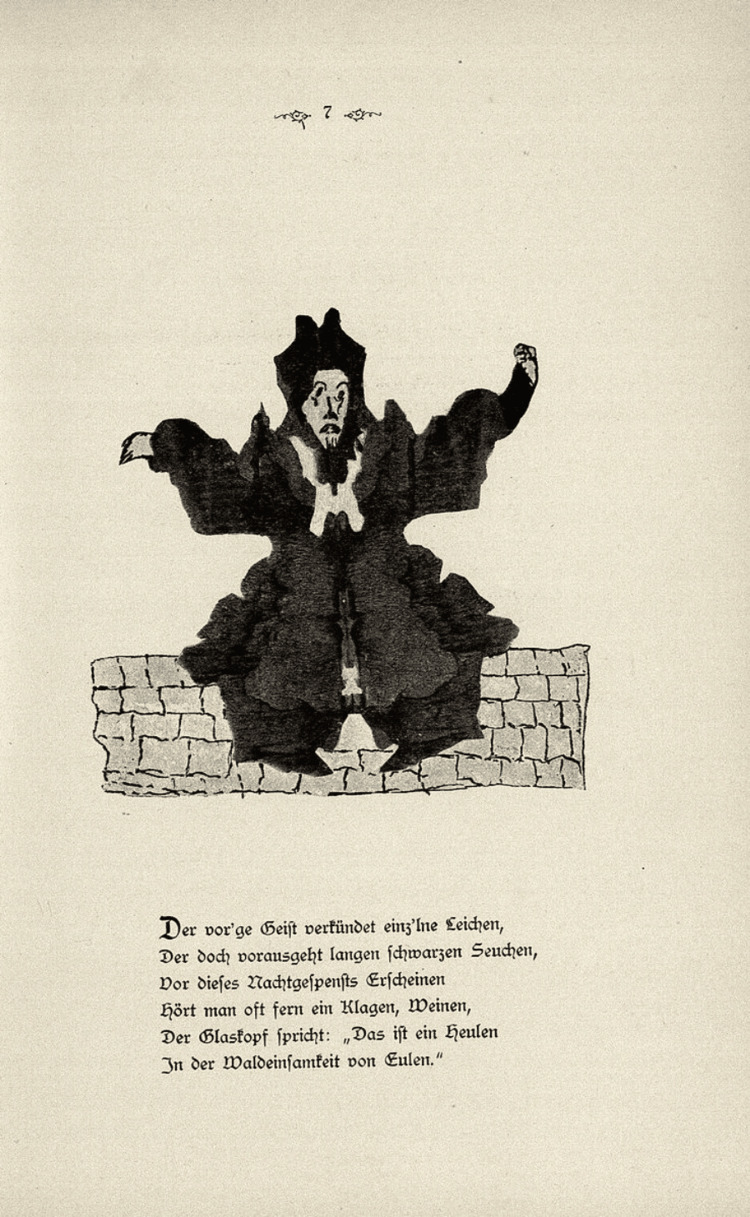
Image from Klexographien by Justinus Kerner Image available on Wikimedia as part of public domain

Hermann Rorschach (Figure 1) was born in 1884 in Zurich, Switzerland, and grew up in a small city called Schaffhausen bordering Germany [3]. Rorschach’s father, Ulrich Rorschach, was a minor European painter and art teacher, who cultivated Rorschach’s artistic character and passion for painting, earning him the nickname ‘klex’ among his peers, the German word for ‘inkblot’ [1,3]. Throughout his life, Rorschach demonstrated a proclivity for *Kleksographie*, or Blotto as it was more commonly known, which was a popular pastime among adolescents that involved filling a sheet of paper with ink and folding it to make various unique figures [4]. Toward the late 1800s, the *Klecksographie* game was first introduced as a new discipline by Justinus Kerner, a German physician and Romantic poet, who published *Klecksographien*, a book of poems with associated inkblots (Figure 2) [1,4]. As early as 1895-1896, Albert Binet considered using inkblots as part of intelligence testing, but eventually abandoned the idea altogether due to administration issues [4,5]. It would not be until some 20 years later that Rorschach, as part of his medical training and education, would create a formal methodology.

Rorschach attended medical school at the University of Zurich, Switzerland, from 1904-1909 at the University Hospital of Psychiatry, Burgholzli [6]. After graduating, Rorschach began his research with inkblots in 1911 with his dissertation entitled, “Reflex Hallucinations and Symbolism,” which would serve as the foundation for Rorschach’s future methodology. In his medical dissertation, Rorschach dealt with the treatment of ‘reflex hallucinations’ which describes a person’s ability to experience a sensory stimulus of one type as the result of a sensory stimulus of another type [1]. An example of a visual perception that led to a tactile-kinesthetic hallucination was in a schizophrenic patient who observed a man using a scythe to cut grass immediately felt as if his own body was being cut [3]. This concept of reflex hallucinations and the resulting crossovers between sensory stimuli ultimately helped Rorschach conceptualize an ideal way of analyzing an individual’s response to an unstructured visual stimulus as represented by inkblots. This would serve as one of the principal tenets of the inkblots in that each individual exerted their preconceived notions or projections onto the stimulus, thus enabling their personal experiences and mental state for extrospection. From 1915-1922, Rorschach worked as a chief psychiatrist at Herisau Hospital, where he was assisted by Hans Behn Eschenburg and created a parallel series of inkblots known as the Behn-Rorschach test.

By 1918, Rorschach had developed 15 inkblots for the purpose of experimentation on the numerous patients at his disposal [7]. This set of inkblots utilized axis and symmetry as their structural elements and were either achromatic (black and white) or chromatic (red, blue, yellow) [7]. Rorschach was insistent on the use of horizontal symmetry around a vertical axis, so that the images would be the same for left and right handed people while also being congruent with how humans perceived bodily symmetry, which in turn gave more emotional physiologic responses [1,8]. This symmetry was also the result of how the images were made by dripping ink onto a sheet of paper and folding it in half to form a complete image [3]. Regarding patient responses, Rorschach developed a scoring system that followed a formal criteria based on individual response to the open ended question, “what might this be?” These criteria included associations based on form (F) and whether the response was based on the whole inkblot (W), a significant detail of the inkblot (D), or a small detail response (Dd) [1,9]. There were also hybrid responses based on the interplay between color and form, allowing for situations where color served as the primary inducer of an image (CF), or vice versa where color served as a secondary role to the shape (FC). One of the most important criteria was movement responses (M), which highlighted whether the patient saw any movement of the inkblot, especially in regard to body parts visualized [3]. Rorschach emphasized movement responses as a key finding regarding schizophrenic patients, who he theorized as having more extreme forms of mental disease and therefore more qualitative modes of altered perception in response to visual stimuli [3,7]. 

**Figure 3:**
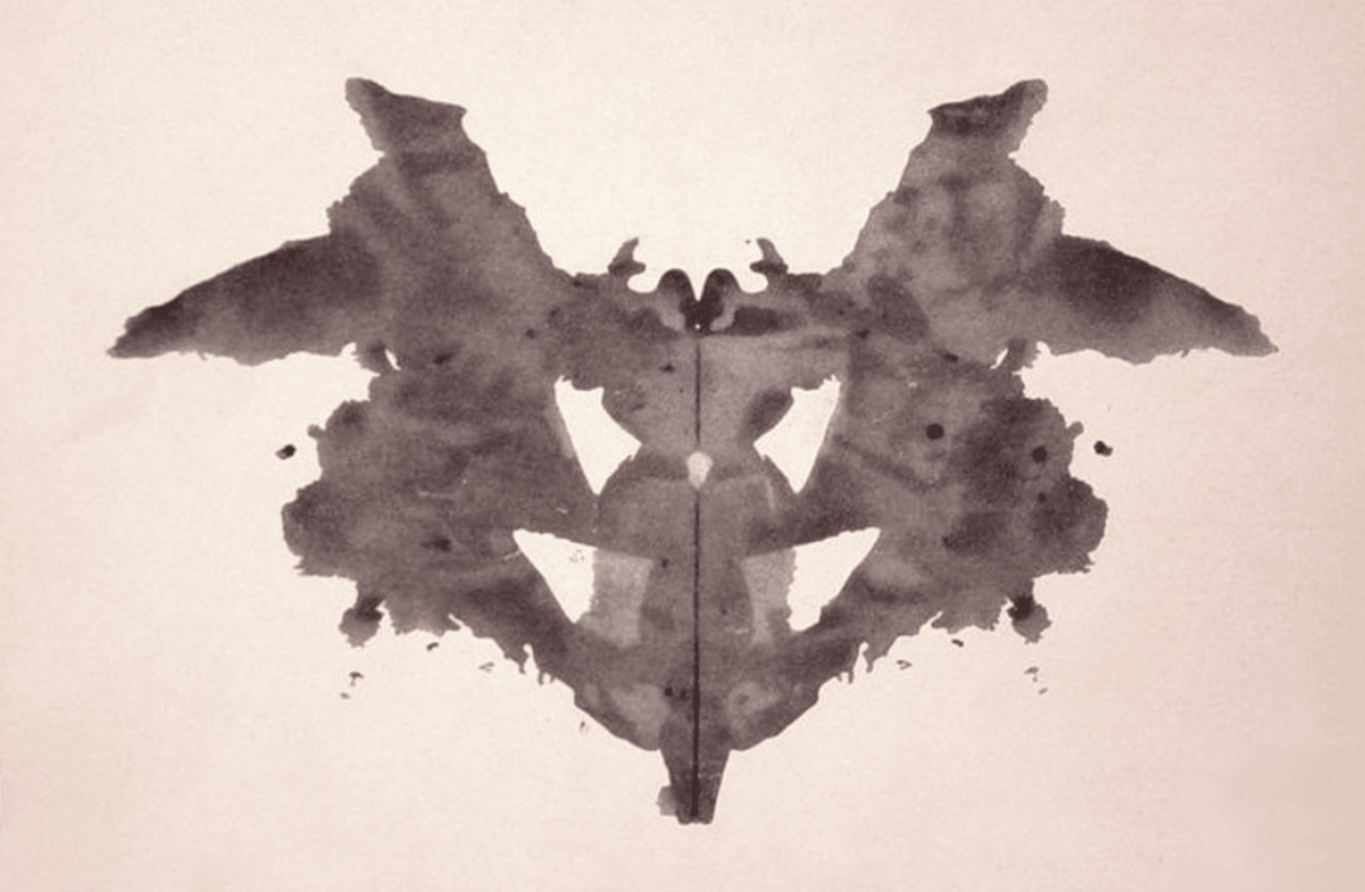
Inkblot No. 1 Image available on Wikimedia as part of public domain

In 1921, Rorschach published his methodology and experimental findings in his landmark book *Psychodiagnostics* along with his final 10 inkblots (Figure 3). The experiment included a modest sum of 405 patients, with 117 serving as normal controls and the other 288 suffering from various mental illnesses that included mania, depression, epilepsy, Korsakoff’s, and the like [9]. Of note, over half of the mental illness group was composed of schizophrenic patients who likely served as the primary focus of Rorschach’s experiment. Although there were no single criteria that was pathognomonic for mental illness, certain patterns emerged between a patient’s disease state and their inkblot responses. Normal patients rarely rejected any cards, contrary to depressive patients who gave few responses and neurotics who frequently rejected cards altogether [1]. Manic patients with an elevated mood often gave multiple whole responses. Schizophrenics often gave Color and Movement answers that were associated with poor form [1]. 

Rorschach ultimately concluded “It is possible by means of the test to draw conclusions concerning many affective relationships [and that] the test has proved to be of diagnostic value" [9]. Rorschach also commented on generalized trends regarding intelligence and personality subtypes, stating “In normals [the test] makes possible differential diagnosis of personality; in patients, the diagnosis of illness. Furthermore, it presents an intelligence test almost completely independent of previous knowledge, memory, practice, and degree of education" [9]. 

The publication of *Psychodiagnostics* celebrated a decade’s worth of research and placed Rorschach at the forefront of the developing field of psychiatry. Yet, for all his work, the Rorschach method was still in its infancy and would require more refinement in the years to come. Unfortunately, just one year later on April 2nd, 1922, Rorschach died due to peritonitis secondary to a ruptured appendix that was mistaken for tobacco poisoning [1,7]. 

## Review

The inkblots after Rorschach’s death

Coming to America (1930s-1940s)

After Rorschach’s death, the inkblots were disseminated to America predominantly through the life work of two men: Samuel Beck (1896-1980) and Bruno Klopfer (1900-1971). While studying psychology at Columbia, Beck was the first to publish American articles on the Rorschach, beginning in 1930 with “Personality Diagnosis by Means of the Rorschach Test,” which investigated the Rorschach test’s “insight into the unconscious mechanisms of personality” in addition to intelligence and emotional stability [1,10]. Bruno Klopfer was a Columbia professor and the first to hold seminars regarding Rorschach that were so popular they necessitated the founding of the *Rorschach Research Exchange* in 1936 for the purpose of disseminating information and ideas related to the test [11]. In 1942, Klopfer published his first major textbook entitled, *The Rorschach Technique*, and like Beck, also commented on the relationship that existed between perception and personality elucidated by the Rorschach technique: “The way in which an individual organizes or ‘structures’ the ink blots in forming his perceptions reflects fundamental aspects of his psychological functioning … [and] reveal such things as the nature of that individual’s inner promptings, his motivations and drive impulses, his capacity to control his impulse, the way he attacks problems, and other aspects of his personality" [12]. 

The emphasis of the Rorschach methodology on perception and personality was not limited to individuals but extended to groups as well as a means of investigating the collective unconscious. The idea of a group Rorschach was first described by an American clinical psychologist, Molly Harrower (1906-1999), who developed a standardized Rorschach multiple choice test for the purpose of psychologically evaluating World War II military recruits [13]. This group approach was promoted to reduce the time required for administration while still providing clinically and statistically relevant information. However, Harrower’s methodology was a departure from Rorschach’s original intent and design that she openly acknowledged: “In this new multiple-choice test…we have departed so far in fact from the essence of what Rorschach intended in the spontaneous unimpeded recording of responses that it is probably fairest to all concern to consider it as an entirely different procedure rather than a further modification of the original method” [13]. Despite the rising popularity of Rorschach testing at the time, the multiple-choice test was never employed by the military. However, the idea of a group Rorschach would not be long forgotten and instead applied to the most notorious group of all: the Nazi defendants at Nuremberg.

The Nazi Rorschachs (1945-1975)

**Figure 4:**
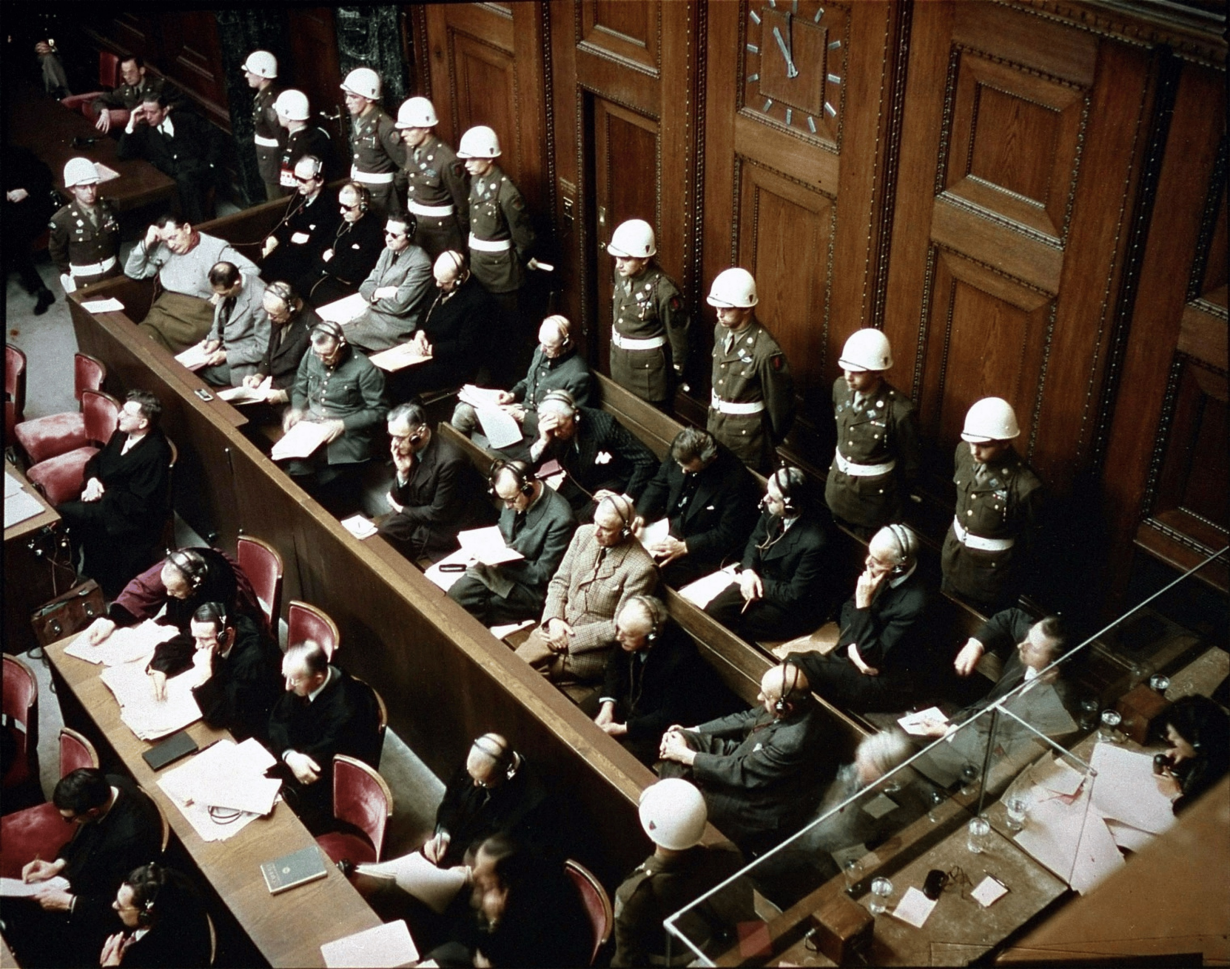
Nazi defendants in the dock at Nuremberg Image available on Wikimedia as part of public domain

The Nuremberg Trials were a series of military tribunals that occurred from November 1945 to October 1946 under the direction of American, European, and Russian forces. Their purpose was to investigate and prosecute 24 members (Figure 4) of the Nazi party who were responsible for a conspiracy to commit crimes against humanity that ultimately resulted in the deaths of 11 million people, the majority of which were Jews. It was against this backdrop that a psychiatrist and Rorschach expert, Douglas Kelly (1912-1958), and an army psychologist, Gustav Gilbert (1911-1977), conducted psychological testing of the Nazi defendants in order to profile the Nazi personality.

Each of the Nazi defendants were administered a Weschler-Bellevue IQ test in addition to the Rorschach inkblot technique [14]. The results, although controversial, are truly mesmerizing. In regard to IQ testing, the mean IQ for 21 Nazi defendants was 128, which fell into the superior to very superior range of intelligence [14]. While several members of the Nazi defendants held doctoral degrees, these results have been met with skepticism since only eight of the 11 subtests were given, excluding those that inquired about general factual information regarding U.S. life in order to minimize cultural bias [14]. 

The Rorschach results were met with even more controversy and skepticism. Kelly presented his paper entitled *Preliminary Report of Rorschach studies of Nazi War Criminals at Nuremberg* that showed the Nazi defendants were anything but special: “From our findings, we must conclude not only that such personalities are not unique or insane, but also that they could be duplicated in any country of the world today. We must also realize that such personalities exist in this country and that there are undoubtedly certain individuals who would willingly climb over the corpses of one half of the people of the United States, if by so doing, they could thereby be given control of the other half” [15]. Gilbert was far more critical and likened the personality type to “the unfeeling, mechanical executioner of order for destruction no matter how horrible, who goes on and on with this ghastly work as though he were a mere machine made of electrical wiring and iron instead of a heart and a mind, with no qualms of conscience or sympathy to restrain him once someone has pressed the button to put him into action with a command” [14]. However, due to publication delays and authorship controversy between Kelly and Gilbert, the Nazi Rorschachs remained unpublished for 30 years. 

In 1975, the Nazi Rorschachs would resurface through a book entitled *The Nuremberg Mind: The Psychology of the Nazi Leaders* by Florence Miale (1916-1990), a Rorschach expert, and Michael Selzer (1940), a writer and political scientist. Miale and Selzer’s book was the first comprehensive book that included the verbatim responses of 16 Nazi defendants that were handed down to them from Gustave Gilbert. Based on their analysis, the frequency of responses demonstrated numerous characteristics and personality traits consistent with psychopaths. Violence was the second most frequent category after depressed mood [16]. This response category was accompanied by numerous Whole responses that favored dark and grotesque imagery that included lowly creatures such as crabs and reptiles, low-level insects such as beetles, and subhuman figures such as devils, gremlins, and trolls [16]. Similarly to Gilbert, Miale and Selzer unequivocally summarized them into a distinctive group: “Our subjects share a number of clinically significant reactions, which individually and in the aggregate, can be considered to set them off as a distinct group. A number of these, and the frequency of their occurrence in this group, point up surprising but nonetheless explicable qualities in the Nazi personality” [16]. However, these hyperbolic results would soon be called into question. 

During this same time, Molly Harrower was also reanalyzing the Nazi Rorschachs and sought to eliminate interpreter bias since the original Rorschach testing and interpretations were performed with full knowledge of the defendant’s identity and historical past [14]. To accomplish this, Harrower selected eight Nazi records and matched them with controls from two different groups: Unitarian ministers and psychiatric outpatients. Harrower then invited 10 Rorschach experts to decide whether or not common denominators or meaningful groups could be formed from their interpretations [17]. Suffice it to say, these 10 experts were unable to detect any common denominator, any group similarities, or group indices of mental disturbance, even when provided with a list of potential groups that included war criminals, members of the clergy, cross-section of middle-class population, patients before and after dialysis, and other groupings [14,17]. In contrast to Miale and Selzer, Harrower’s findings echoed those of Kelly 30 years prior in that there was nothing significant regarding the Nazi defendants or their personalities.

A United Rorschach Methodology: John Exner and His ‘Controversial’ Comprehensive System (1970s)

Over a period of roughly 20 years (1936-1957), five American Rorschach systems were developed that, although not completely different from each other, defied comparison due to issues of scoring and approach to interpretation [4]. These methods were finally united in 1974 by clinical psychologist John Exner when he published *The Rorschach: A Comprehensive System*. Exner’s System was the first to standardize the method for administering the test and added several new scoring indices and formulas in an effort to objectively quantify the responses. This approach made the *Comprehensive System* more nuanced and complex and added nearly 140 new variables to Rorschach’s original dozen or so codes [1]. However, Exner’s effort to objectify and standardize the approach also ultimately led to its demise by making it easier to criticize and subject to controversy. An analysis performed by Vincent and Harman (1991) showed that less than one-fifth of Exner’s variables were clinically significant [18]. The most detrimental evidence came from meta-analysis examining the clinical validity of the large number of the Exner variables. One study by Mihura et al. reported for 65 main Exner variables, only 13 were found to have excellent support (r>.33, p < 00.1) [19]. Another meta-analysis performed by Wood et al. examined the validity of 37 Rorschach variables to discriminate psychopaths from non-psychopaths and reported only five variables exhibited a modest relationship with psychopathy [20]. For these reasons, the Rorschach test has largely fallen out of favor and is rarely performed today.

## Conclusions

Like his enigmatic inkblots, the legacy of Rorschach is complex and does not represent a single entity per se, but rather the amalgamation of experiences that range across time from Rorschach to other independent investigators. The inkblots, which have remained unchanged for over a century, are certainly representative of Rorschach’s life and upbringing as an artist, but also as a psychiatrist. However, the Rorschach methodology is not without its controversies. Their application during the Nuremberg Trials demonstrated questionable results regarding the Nazi personality due to interpreter bias as elucidated by Harrower. Several meta-analysis results regarding Exner’s Comprehensive System have shown questionable validity as well as a lack of clinical relevance. Despite these controversies, the legacy of the Rorschach inkblots lives on having continually captivated and romanced the collective psyche of modern society, appearing in movies such as The Dark Mirror, in modern art with Andy Warhol’s series of Rorschach paintings, and in pop culture with the vigilante Watchmen comic book character Rorschach. Ultimately, Rorschach’s inkblots represent mankind’s attempt to understand the human psyche and the man behind the inkblot who dared to ask, “what might this be?”
